# Fabrication-constrained nanophotonic inverse design

**DOI:** 10.1038/s41598-017-01939-2

**Published:** 2017-05-11

**Authors:** Alexander Y. Piggott, Jan Petykiewicz, Logan Su, Jelena Vučković

**Affiliations:** Ginzton Laboratory, Stanford University, Stanford, California 94305 USA

## Abstract

A major difficulty in applying computational design methods to nanophotonic devices is ensuring that the resulting designs are fabricable. Here, we describe a general inverse design algorithm for nanophotonic devices that directly incorporates fabrication constraints. To demonstrate the capabilities of our method, we designed a spatial-mode demultiplexer, wavelength demultiplexer, and directional coupler. We also designed and experimentally demonstrated a compact, broadband 1 × 3 power splitter on a silicon photonics platform. The splitter has a footprint of only 3.8 × 2.5 *μ*m, and is well within the design rules of a typical silicon photonics process, with a minimum radius of curvature of 100 nm. Averaged over the designed wavelength range of 1400–1700 nm, our splitter has a measured insertion loss of 0.642 ± 0.057 dB and power uniformity of 0.641 ± 0.054 dB.

## Introduction

Nanophotonic devices are typically designed by starting with an analytically designed structure, and hand-tuning a few parameters^1^. In recent years, it has become increasingly popular to automate this process with the use of powerful optimization algorithms. In particular, by searching the full space of possible structures, it is possible to design devices with higher performance and smaller footprints than traditional devices^2–8^.

A major challenge when designing devices with arbitrary topologies is ensuring that the structures remain fabricable. Many of these computationally designed structures have excellent performance when fabricated using high-resolution electron-beam lithography, but they have features which are difficult to resolve with industry-standard optical lithography^3, 7, 8^.

Building on our previous work^5, 7, 9^, we propose an inverse design method for nanophotonic devices that incorporates fabrication constraints. Our algorithm achieves an approximate minimum feature size by imposing curvature constraints on dielectric boundaries in the structure. We then demonstrate the capabilities of our method by designing a spatial-mode demultiplexer, wavelength demultiplexer, and directional coupler, and experimentally demonstrating an ultra-broadband 1 × 3 power splitter. All of our designs are compact, have no small features, and should be resolvable using modern photolithography. Additionally, with the exception of the wavelength demultiplexer, all of our devices are well within the design rules of existing silicon photonics processes.

## Design Method

Due to the complexity of accurately modelling lithography and etching processes, most attempts to incorporate fabrication constraints into computational nanophotonic design have focused on heuristic methods. One approach is to restrict the design to rectangular pixels which are larger than the mininum allowable feature size^10^. The resulting Manhattan geometry, however, is restrictive and likely not optimal for optical devices. Another method involves applying a convolutional filter to the design followed by thresholding^11–13^, which can introduce artifacts smaller than the desired feature size. The approach used in this work is to impose curvature constraints on the device boundaries, which avoids the aforementioned issues. Curvature limits have been successfully applied in earlier work^4^, but were not described in detail nor validated with experimental demonstrations.

### Level Set Formulation

We assume that our device is planar and consists of only two materials. We can represent our structure by constructing a continuous function $\varphi (x,y):{{\mathbb{R}}}^{2}\to {\mathbb{R}}$ over our design region, and letting the boundaries between the materials lie on the level set *ϕ* = 0. The permittivity *ε* is then given by

1 $$\varepsilon (x,y)=\{\begin{array}{ll}{\varepsilon }_{1} & {\rm{for}}\,\varphi (x,y)\le 0\\ {\varepsilon }_{2} & {\rm{for}}\,\varphi (x,y) > 0.\end{array}$$

The advantage of this implicit representation is that changes in topology, such as the merging and splitting of holes, are trivial to handle. We can also manipulate our structure by adding a time dependence, and evolving *ϕ*(*x*, *y*, *t*) as a function of time *t* with a variety of partial differential equations collectively known as level set methods^14, 15^.

To design a device, we first choose some objective function *f* [*ε*] which describes how well the structure matches our electromagnetic performance constraints^5, 7^. We then evolve our structure, represented by *ϕ*, in such a way that we minimize our objective *f*. We can achieve this by adapting gradient descent optimization to our level set representation. The level set equation for moving boundaries in the normal direction is

2 $${\varphi }_{t}+v(x,y)|\nabla \varphi |=0$$

where $\nabla \varphi ={\varphi }_{x}+{\varphi }_{y}$ is the spatial gradient of *ϕ*, and *v*(*x*, *y*) is the local velocity. To implement gradient descent, we choose the velocity field *v*(*x*, *y*) to correspond to the gradient of the objective function *f* [*ε*]^15^. The gradient can be efficiently computed using adjoint sensitivity analysis^3, 4, 6, 9^. As *t* → ∞, *ϕ* converges to a locally optimal structure.

Unfortunately, this approach tends to result in the formation of extremely small features. We can avoid this problem by periodically enforcing curvature constraints. The level set equation for smoothing out curved regions is

3 $${\varphi }_{t}-\kappa |\nabla \varphi |=0$$

where the local curvature *κ* is given by

4 $$\kappa =\nabla \cdot (\frac{\nabla \varphi }{|\nabla \varphi |})=\frac{{\varphi }_{x}^{2}{\varphi }_{yy}-2{\varphi }_{x}{\varphi }_{y}{\varphi }_{xy}+{\varphi }_{xx}{\varphi }_{y}^{2}}{{|\nabla \varphi |}^{3}}.$$

Although equation (3) removes highly curved regions more quickly^14^, the boundaries are eventually reduced to a set of straight lines with zero curvature as *t* → ∞.

From a fabrication perspective, we only need to smooth regions which are above some maximum allowable curvature *κ*_0_. We can do this by introducing a weighting function

5 $$b(\kappa )=\{\begin{array}{ll}1 & {\rm{for}}\,|\kappa | > {\kappa }_{0}\\ 0 & {\rm{otherwise}}\end{array}$$

and modifying equation (3) to be

6 $${\varphi }_{t}-b(\kappa )\kappa |\nabla \varphi |=0.$$

If we evolve *ϕ* with equation (6) until it reaches steady state, the maximum curvature will be less than or equal to *κ*_0_.

Although curvature limiting will eliminate the formation of most small features, it does not prevent the formation of narrow gaps or bridges. We detect these features by applying morphological dilation and erosion operations to the set *ϕ* > 0, and checking for changes in topology. Once detected, these narrow gaps and bridges can be eliminated by “cutting” them in half, and then applying curvature filtering to round out the sharp edges.

The final design algorithm is as follows:

1. Initialize *ϕ* and *δt*.
2. Repeat until *δt* < *δt*_*min*_.

- Let *ϕ*′ ← *ϕ*.
- **Gradient descent**: evolve *ϕ*′ with eqn. (2) for time *δt*.
- **Gap and bridge removal**: detect any small gaps or bridges, and modify *ϕ*′ to remove them.
- **Curvature limit**: evolve *ϕ*′ with eqn. (6) until convergence.
- **If** *f*[*ε*[*ϕ*′]] < *f*[*ε*[*ϕ*]], **then** let *ϕ* ← *ϕ*′ and increase *δt*.

**Otherwise**, decrease *δt*.

A detailed description of the objective function *f*[*ε*] and implementation details can be found in the supplementary information.

## Designed Devices

To demonstrate the capabilities of our design method, we designed a variety of three-dimensional waveguide-coupled devices on a silicon photonics platform. All of the structures we show here consist of a single fully-etched 220 nm thick Si layer with SiO_2_ cladding. Refractive indices of *n*_Si_ = 3.48 and ${n}_{{{\rm{SiO}}}_{{\rm{2}}}}=1.44$ were used.

### 1 × 3 splitter

Our first device is a broadband 1 × 3 power splitter with 500 nm wide input and output waveguides. We constrained the minimum radius of curvature to be 100 nm, well within the typical design rules of a silicon photonics process, and enforced bilateral symmetry. To design the splitter, we specified that power in the fundamental traverse-electric (TE) mode of the input waveguide should be equally split into the fundamental TE mode of the three output waveguides, with at least 95% efficiency. Broadband performance was achieved by simultaneously optimizing at 6 equally spaced wavelengths from 1400–1700 nm.

**Figure 1:**
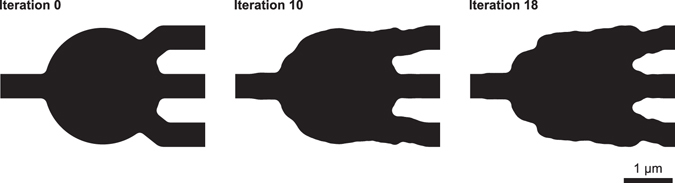
Intermediate steps in the optimization process for the 1 × 3 splitter. Starting with a star-shaped geometry, the optimization converged in 18 iterations. The minimum radius of curvature in the design was set to 100 nm. Regions with Si are denoted in black, and SiO_2_ is denoted in white.

The optimization process is illustrated in Fig. 1; the simulated fields and performance are presented later in this paper alongside experimental results. Starting with a star-shaped geometry, the optimization process converged in 18 iterations. Each iteration required two electromagnetic simulations per design frequency (see supplementary information), resulting in a total of 216 simulations. The device was designed in approximately 2 hours on a single server with an Intel Core i7-5820 K processor, 64 GB of RAM, and three Nvidia Titan Z graphics cards. Since the computational cost of optimization is dominated by the electromagnetic simulations, we performed them using a graphical processing unit (GPU) accelerated implementation of the finite-difference frequency-domain (FDFD) method^16, 17^, with a spatial step size of 40 nm. A single FDFD solve is considerably faster and less computationally expensive than a finite-difference time-domain (FDTD) simulation.

Interestingly, the splitter appears to be operate using the multi-mode interferometer (MMI) principle^18^, with a geometry that resembles a boundary-optimized MMI. This was without any human input or intervention throughout the design process, suggesting that MMI-based devices may be optimal for this particular application.

### Spatial mode demultiplexer

Our second device is a spatial-mode demultiplexer that takes the TE_10_ and TE_20_ modes of a 750 nm wide input waveguide, and routes them to the fundamental TE mode of two 400 nm wide output waveguides. To design this device, we specified that >90% of the input power should be transmitted to the desired output port, and <1% should be coupled into the other output. As with the 1 × 3 splitter, this device was designed to be broadband by optimizing at six evenly spaced wavelengths between 1400 nm and 1700 nm. To obtain an initial structure for the level set optimization, we started with a uniform permittivity in the design region, allowed the permittivity to vary continuously in the initial stage of optimization, and applied thresholding to obtain a binary structure^5^. We used a minimum radius of curvature of 70 nm, and a minimum gap or bridge width of 90 nm.

**Figure 2:**
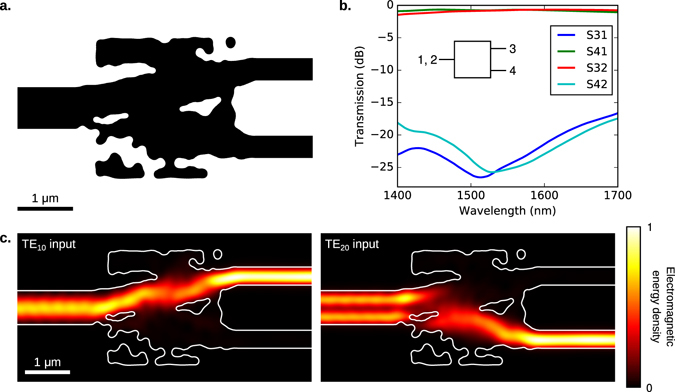
A spatial mode demultiplexer that takes the TE_10_ and TE_20_ modes of a 750 nm wide input waveguide, and routes them to the TE_10_ mode of two 400 nm wide output waveguides. Here, we present (**a**) the final design, (**b**) simulated S-parameters, and (**c**) the electromagnetic energy density $U=\frac{1}{2}\varepsilon {E}^{2}+\frac{1}{2}\mu {H}^{2}$ at 1550 nm, where *ε* and *μ* are the permittivity and permeability, and *E* and *H* are the electric and magnetic fields respectively. The fields and S-parameters were calculated using finite-difference time-domain (FDTD) simulations. The boundaries of the device are outlined in white.

The final design and simulated performance are illustrated in Fig. 2. The spatial mode multiplexer has an average insertion loss of 0.826 dB, and a contrast better than 16 dB over the design bandwidth of 1400–1700 nm.

### Wavelength demultiplexer

Our third device is a 3-channel wavelength demultiplexer with a 40 nm channel spacing with 500 nm wide input and output waveguides. To design this device, we specified that >80% of the input power should be transmitted to the desired output port, and <1% should be coupled into the remaining outputs. The initial structure was a rectangular slab of silicon with a regular array of holes, which had a pitch of 400 nm and a diameter of 250 nm. We enforced a minimum radius of curvature of 40 nm, and a minimum gap or bridge width of 90 nm.

**Figure 3:**
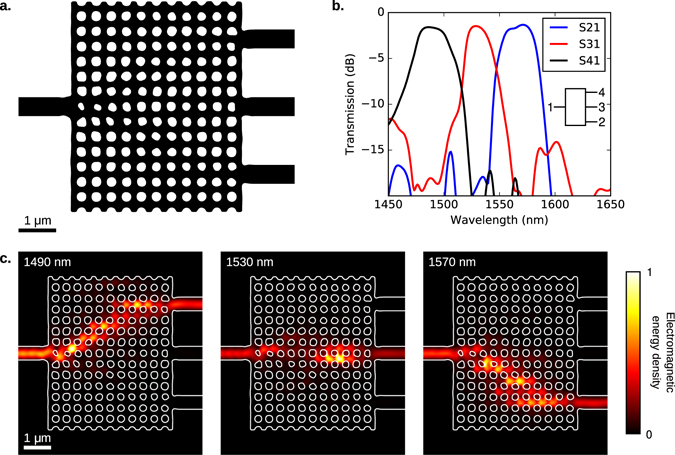
A compact 3-channel wavelength demultiplexer with a 40 nm channel spacing. The input and output waveguides are all 500 nm wide. Here, we present (**a**) the final design, (**b**) simulated S-parameters, and (**c**) the electromagnetic energy density at the three operating wavelengths.

The final design and simulated performance are illustrated in Fig. 3. At the center of each channel, the insertion loss is approximately 1.5 dB, and the contrast is better than 16 dB. Each channel has a usable bandwidth >10 nm.

#### Directional coupler

Our final device is a relatively compact 50-50 directional coupler, with 400 nm input and output waveguides. This device was designed by specifying that half the power in fundamental mode of the input waveguide should be coupled into each of the outputs, with >90% efficiency. As in the design of the spatial mode demultiplexer, we obtained an initial structure by starting with a uniform permittivity in the design region, allowing the permittivity to vary continuously in the initial stage of optimization, and applying thresholding. To achieve moderate broadband performance, the device was simultaneously optimized for 6 wavelengths between 1470–1630 nm. We enforced a minimum radius of curvature of 70 nm, and a minimum bridge width of 90 nm.

**Figure 4:**
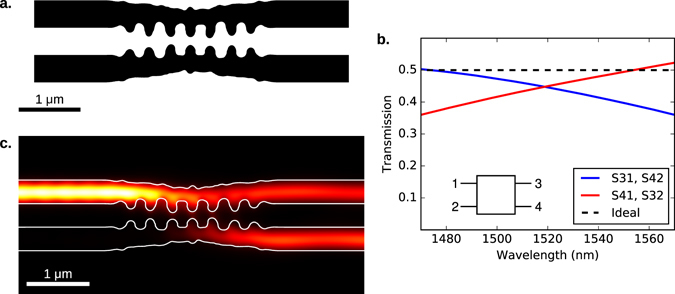
A relatively compact and broadband 50–50 directional coupler with 400 nm input and output waveguides. The device resembles a grating-assisted directional coupler. Here, we present (**a**) the final design, (**b**) simulated S-parameters, and (**c**) the electromagnetic energy density at 1550 nm.

The final device and simulated performance are illustrated in Fig. 4. At the optimal operating point of 1520 nm, the device couples 90% of the input power into the desired output waveguides. The device structure appears to be a grating-assisted directional coupler.

## Experimental Realization of 1 × 3 Splitter

Robust and efficient power splitters are essential building blocks for integrated photonics. A variety of 1 × 2 splitters with attractive performance have been demonstrated on the silicon photonics platform, ranging from conventional devices^19, 20^ to those designed using advanced optimization techniques^4, 21, 22^. However, it is not possible to split power equally into an arbitrary number of waveguides by cascading 1 × 2 splitters, and efficient and compact devices that fill this gap are lacking in the literature. To help fill this gap, we fabricated and experimentally demonstrated the 1 × 3 splitter we presented in the previous section. Our 1 × 3 splitter is considerably smaller and more broadband than any existing device in the literature^23, 24^.

### Fabrication

The power splitters were fabricated on Unibond SmartCut silicon-on-insulator (SOI) wafers obtained from SOITEC, with a nominal 220 nm device layer, and 3.0 *μ*m buried oxide layer. A JEOL JBX-6300FS electron-beam lithography system was used to pattern a 330 nm thick layer of ZEP-520A resist spun on the samples. A transformer-coupled plasma etcher was used to transfer the pattern to the device layer, using a C_2_F_6_ breakthrough step and BCl_3_/Cl_2_/O_2_ main etch. The mask was stripped by soaking in solvents, followed by a piranha (H_2_SO_4_/H_2_O_2_) clean. Finally, the devices were capped with 1.6 *μ*m of LPCVD (low pressure chemical vapour deposition) oxide.

A multi-step etch-based process was used to expose waveguide facets for edge coupling. First, a chrome mask was deposited using liftoff to protect the devices. Next, the oxide cladding, device layer, and buried oxide layer were etched in a inductively-coupled plasma etcher using a C_4_F_8_/ArO_2_ chemistry. To provide mechanical clearance for the optical fibers, the silicon substrate was then etched to a depth of ~100 *μ*m using the Bosch process in a deep reactive-ion etcher (DRIE). Finally, the chrome mask was chemically stripped, and the samples were diced into conveniently-sized pieces.

### Characterization

**Figure 5:**
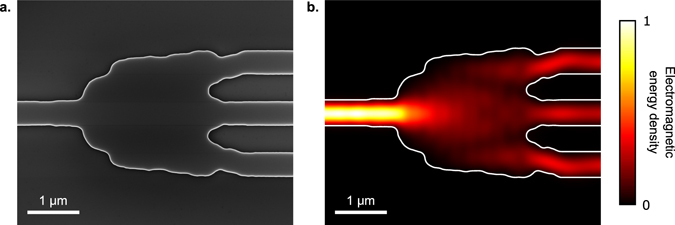
The broadband 1 × 3 splitter. (**a**) SEM image of the fabricated splitter. The device was made by fully etching the 220 nm device layer of an SOI wafer. The total footprint is 3.8 × 2.5 *μ*m. This image was taken before the devices were capped with oxide. (**b**) Electromagnetic energy density in the device at 1550 nm.

The final splitter is illustrated in Fig. 5, showing both an scanning-electron micrograph (SEM) of the fabricated device, and simulated electromagnetic energy density at the center wavelength of 1550 nm. The simulated electric energy density ${U}_{E}=\frac{1}{2}\varepsilon {E}^{2}$ as a function of wavelength is shown in the supplementary video.

Transmission through the device was measured by edge-coupling to the input and output waveguides using lensed fibers. A polarization-maintaining fiber was used on the input side to ensure that only the TE mode of the waveguide was excited. To obtain consistent coupling regardless of the transmission spectra of the devices, the fibers were aligned by optimizing the transmitted power of a 1570 nm laser. The transmission spectrum was then measured by using a supercontinuum source and a spectrum analyzer. The device characteristics were obtained by normalizing the transmission with respect to a waveguide running parallel to the device.

**Figure 6:**
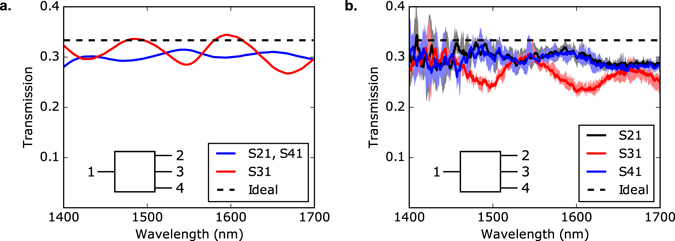
Simulated and measured S-parameters for the broadband 1 × 3 splitter, where *Sij* is the transmission from port *i* to port *j*. (**a**) Simulated performance, calculated using finite-difference time-domain (FDTD) simulations. Due to bilateral symmetry in the structure, S21 and S41 are equal to each other. (**b**) Measured device performance. Here, we have overlaid the measurements for 4 identically fabricated devices. The average values are denoted by the solid lines, and the minimum and maximum values are denoted by the shaded areas.

The simulated and measured transmission spectra of the device are plotted in Fig. 6. The simulations and measurements match reasonably well, although the measured devices have slightly higher losses and exhibit a spectral shift with respect to simulations. The device performance is highly consistent across all 4 measured devices, indicating that they are robust to fabrication error. The spectral shifts are likely due to slight over-etching or under-etching errors, as indicated by simulations we present in the supplementary information.

The two key criteria for a power splitter are low insertion loss, and excellent power uniformity. The power uniformity is defined as the ratio between the maximum and minimum output powers. Averaged over the designed wavelength range of 1400–1700 nm, our 1 × 3 splitter has a measured insertion loss of 0.642 ± 0.057 dB, and a power uniformity of 0.641 ± 0.054 dB. Here, the uncertainty refers to the variability between different measured devices.

## Conclusion

In summary, we have incorporated fabrication constraints into an inverse design algorithm for nanophotonic devices. Using this method, we designed a spatial mode demultiplexer, a 3-channel wavelength demultiplexer, and 50–50 directional coupler. We also designed and experimentally demonstrated a broadband 1 × 3 splitter. Critically, our devices have no small features which would be difficult to resolve with photolithography, paving the way for inverse designed structures to become practical components of integrated photonics systems.

## Electronic supplementary material

Supplementary video

Supplementary information

## References

[1] Reed, G. T. *Silicon Photonics: The State of the Art* (John Wiley & Sons, Chichester, West Sussex, U.K. 2008).

[2] Mutapcica A, Boyd S, Farjadpour A, Johnson SG, Avnielb Y (2009). Robust design of slow-light tapers in periodic waveguides. Eng. Optimiz..

[3] Jensen JS, Sigmund O (2011). Topology optimization for nano-photonics. Laser Photonics Rev.

[4] Lalau-Keraly CM, Bhargava S, Miller OD, Yablonovitch E (2013). Adjoint shape optimization applied to electromagnetic design. Opt. Express.

[5] Lu J, Vučković J (2013). Nanophotonic computational design. Opt. Express.

[6] Niederberger ACR, Fattal DA, Gauger NR, Fan S, Beausoleil RG (2014). Sensitivity analysis and optimization of sub-wavelength optical gratings using adjoints. Opt. Express.

[7] Piggott AY (2015). Inverse design and demonstration of a compact and broadband on-chip wavelength demultiplexer. Nature Photonics.

[8] Frellsen LF, Ding Y, Sigmund O, Frandsen LH (2016). Topology optimized mode multiplexing in silicon-on-insulator photonic wire waveguides. Opt. Express.

[9] Piggott AY (2014). Inverse design and implementation of a wavelength demultiplexing grating coupler. Sci. Rep..

[10] Shen B, Wang P, Polson R, Menon R (2015). An integrated-nanophotonics polarization beamsplitter with 2.4 × 2.4 *μ*m footprint. Nature Photonics.

[11] Elesin Y, Lazarov B, Jensen J, Sigmund O (2012). Design of robust and efficient photonic switches using topology optimization. Phot. Nano. Fund. Appl..

[12] Deng Y, Korvink JG (2016). Topology optimization for three-dimensional electromagnetic waves using an edge element-based finite-element method. Proc. R. Soc. A.

[13] Frandsen LH, Sigmund O (2016). Inverse design engineering of all-silicon polarization beam splitters. Proc. SPIE.

[14] Osher, S. & Fedkiw, R. *Level Set Methods and Dynamic Implicit Surfaces* (Springer, New York, USA 2003).

[15] Burger M, Osher SJ (2005). A survey on level set methods for inverse problems and optimal design. Eur. J. Appl. Math..

[16] Shin W, Fan S (2012). Choice of the perfectly matched layer boundary condition for frequency-domain Maxwell’s equations solvers. J. Comput. Phys..

[17] Shin W, Fan S (2013). Accelerated solution of the frequency-domain Maxwell’s equations by engineering the eigenvalue distribution. Opt. Express.

[18] Besse PA, Bachmann M, Melchior H, Soldano LB, Smit MK (1994). Optical bandwidth and fabrication tolerances of multimode interference couplers. J. Lightwave Technol..

[19] Sakai, A., Fukuzawa, T. & Baba, T. Low loss ultra-small branches in a silicon photonic wire waveguide. *IEICE Trans. Electron*. E85-C, 1033–1038 (2002).

[20] Tao SH (2008). Cascade wide-angle y-junction 1 × 16 optical power splitter based on silicon wire waveguides on silicon-on-insulator. Opt. Express.

[21] Borel PI (2005). Topology optimised broadband photonic crystal y-splitter. Electron. Lett..

[22] Zhang Y (2013). A compact and low loss y-junction for submicron silicon waveguide. Opt. Express.

[23] Besse PA, Gini E, Bachmann M, Melchior H (1996). New 2 × 2 and 1 × 3 multimode interference couplers with free selection of power splitting ratios. J. Lightwave Technol..

[24] Zhang M, Malureanu R, Krüger AC, Kristensen M (2010). 1 × 3 beam splitter for te polarization based on self-imaging phenomena in photonic crystal waveguides. Opt. Express.

