# Supplementary material for: Fabrication-constrained nanophotonic inverse design
Source: Sci Rep. 2017 May 11;7:1786. doi: 10.1038/s41598-017-01939-2 (PMC5431915; doi:10.1038/s41598-017-01939-2)
Supplement: Supplementary file 2 — Supplementary information [file 41598_2017_1939_MOESM2_ESM.pdf]

Supplementary Information:
Fabrication-constrained nanophotonic inverse design

Alexander Y. Piggott, Jan Petykiewicz, Logan Su, and Jelena Vučković

1 Electromagnetic Design Method

1.1 Problem description

Our goal is to automate the design of all passive photonic structures. Thus, our first task is to come up with a generic way of defining the functionality of a optical device. One approach is to describe the coupling between a set of input and output modes, since any linear optical device can be described in this fashion [1]. This is particularly useful for waveguide-coupled devices, whose functionality can be defined in terms of the guided modes of the input and output waveguides.

In our design method, we specify device functionality by describing the mode conversion efficiency between a set of input modes and output modes. The input and output modes are specified by the user, and kept fixed during the optimization process. The input modes $i = 1 \ldots M$ are at frequencies ω_i, and can be represented by an equivalent current density distribution $\mathbf{J}_i$. The fields $\mathbf{E}_i$ produced by each input mode satisfy Maxwell's equations,

$$\nabla \times \mu_0^{-1} \nabla \times \mathbf{E}_i - \omega^2 \epsilon \mathbf{E}_i = -i\omega_i \mathbf{J}_i \tag{S.1}$$

where ϵ is the permittivity distribution, and μ_0 is the permeability of free space.

For each input mode i, we then specify a set of output modes $j = 1 \ldots N_i$, whose amplitudes are bounded between α_{ij} and β_{ij}. If our output modes are guided modes of waveguides with modal electric fields $\mathcal{E}_{ij}$ and magnetic fields $\mathcal{H}_{ij}$, this constraint can be written using a mode orthogonality relationship [2],

$$\alpha_{ij} \leq \left| \iint (\mathbf{E}_i \times \mathcal{H}_{ij} + \mathcal{E}_{ij} \times \mathbf{H}_i) \cdot \hat{n} \, d\mathbf{r}_\perp \right| \leq \beta_{ij}. \tag{S.2}$$

Here, $\hat{n}$ is a unit vector pointing in the propagation direction, and $\mathbf{r}_\perp$ denotes the coordinates perpendicular to the propagation direction. We can use Faraday's law $\nabla \times \mathbf{E}_i = -i\omega\mu_0 \mathbf{H}_i$ to rewrite (S.2) purely in terms of the electric field:

$$\alpha_{ij} \leq \left| \iint \left(\mathbf{E}_i \times \mathcal{H}_{ij} + \mathcal{E}_{ij} \times \frac{i}{\omega\mu_0} \nabla \times \mathbf{E}_i \right) \cdot \hat{n} \, d\mathbf{r}_\perp \right| \leq \beta_{ij}. \tag{S.3}$$

More generally, we can specify the output mode amplitude in terms of a linear functional $\mathcal{L}_{ij}$ of the electric field $\mathbf{E}_i$,

$$\alpha_{ij} \leq \left|\mathcal{L}_{ij}\left(\mathbf{E}_i\right)\right| \leq \beta_{ij}, \tag{S.4}$$

where $V = \{\mathbf{E} : \mathbb{R}^3 \to \mathbb{C}^3\}$ is the space of all possible electric field distributions, and $\mathcal{L}_{ij} : V \to \mathbb{C}$ maps electric field distributions to a complex scalar.

We are thus interested in finding a permittivity distribution ϵ and electric fields $\mathbf{E}_i$ which simultaneously satisfy (S.1) and (S.4), for all input modes $i = 1 \dots M$ and output modes $j = 1 \dots N$. To ensure that the resulting device is fabricable, we will later impose additional constraints on ϵ.

1.2 Linear algebra description

Since we will solve Maxwell's equations numerically, and employ numerical optimization techniques to design our devices, it is convenient to recast the design problem in terms of linear algebra. We do this by discretizing space and making the substitutions

$$\begin{aligned} \mathbf{E}_i &\to x_i \in \mathbb{C}^n \\ \epsilon &\to z \in \mathbb{C}^n \\ \nabla \times \mu_0^{-1} \nabla \times &\to D \in \mathbb{C}^{n \times n} \\ -i\omega_i \mathbf{J}_i &\to b_i \in \mathbb{C}^n \\ \mathcal{L}_{ij} &\to c_{ij} \in \mathbb{C}^n. \end{aligned} \tag{S.5}$$

We thus wish to find electric fields x_i and a permittivity distribution z which satisfy

$$Dx_i - \omega_i^2 \operatorname{diag}(z) x_i - b_i = 0 \tag{S.6}$$

$$\alpha_{ij} \leq \left|c_{ij}^\dagger x_i\right| \leq \beta_{ij} \tag{S.7}$$

for $i = 1 \dots M$ and $j = 1 \dots N_i$. Here, $\operatorname{diag}(v)$ refers to the diagonal matrix whose diagonal entries are given by the vector v, and $u^\dagger$ is the conjugate transpose of u. For convenience, we further define the matrices

$$\begin{aligned} A_i(z) &= D - \omega_i^2 \operatorname{diag}(z) \\ B_i(x_i) &= -\omega_i^2 \operatorname{diag}(x_i). \end{aligned} \tag{S.8}$$

This lets us rewrite equation (S.6) as

$$0 = A_i(z)x_i - b_i = B_i(x_i)z + \left(Dx_i - b_i\right). \tag{S.9}$$

The final problem we wish to solve is then

$$A_i(z)x_i - b_i = 0 \tag{S.10}$$

$$\alpha_{ij} \leq \left|c_{ij}^\dagger x_i\right| \leq \beta_{ij} \tag{S.11}$$

for $i = 1 \dots M$ and $j = 1 \dots N_i$.

1.3 Parametrizing the structure

As described in the main article, we describe our structure using a two-dimensional level-set function $\phi(x, y) : \mathbb{R}^2 \to \mathbb{R}$, where the permittivity in the design region is given by

$$\epsilon(x, y) = \begin{cases} \epsilon_1 & \text{for } \phi(x, y) \leq 0 \\ \epsilon_2 & \text{for } \phi(x, y) > 0. \end{cases} \tag{S.12}$$

For the purposes of numerical optimization, we discretize the level set function in space, which transforms the level set function into a two dimensional array $\phi \in \mathbb{R}^{U \times V}$.

We parametrize the permittivity distribution z with the level set ϕ by using a mapping function $m : \mathbb{R}^{U \times V} \to \mathbb{C}^n$, where

$$z = m(\phi). \tag{S.13}$$

When the level set boundaries are not perfectly aligned with simulation grid cells, we render the structure using anti-aliasing. This allows us to continuously vary the structure, rather than being forced to make discrete pixel-by-pixel changes.

1.4 Formulating the optimization problem

We are finally in a position to construct our optimization problem. Although there are a variety of ways we could solve (S.10) and (S.11), the particular optimization problem we choose to solve is

$$\begin{array}{ll} \text{minimize} & F(x_1, \ldots, x_M) \\ \text{subject to} & A_i(z)x_i - b_i = 0, \quad \text{for } i = 1 \ldots N_i \\ & z = m(\phi). \end{array} \tag{S.14}$$

Here, we constrain the fields to satisfy Maxwell's equations, parameterize the permittivity z with the level set function $\phi \in \mathbb{R}^{U \times V}$, and construct a penalty function

$$F(x_1, \ldots, x_M) = \sum_{i=1}^{M} f_i(x_i) \tag{S.15}$$

for violating our field constraints from equation (S.11). The penalty $f_i(x_i)$ for each input mode is given by

$$f_i = \sum_{j=1}^{N_i} I_+\left(\left|c_{ij}^\dagger x_i\right| - \alpha_{ij}\right) + I_+\left(\beta_{ij} - \left|c_{ij}^\dagger x_i\right|\right) \tag{S.16}$$

where $I_+(u)$ is a relaxed indicator function [3],

$$I_+(u) = \begin{cases} 0, & u \geq 0 \\ \frac{1}{s}|u|^q, & \text{otherwise.} \end{cases} \tag{S.17}$$

Typically, we use $q = 2$ and $s = \max_i f_i(x_i)$.

1.5 Steepest descent optimization

We solve our optimization problem (S.14) by first ensuring that Maxwell's equations (S.10) are always satisfied. This implies that both the fields $x_1, \ldots, x_M$ and the field-constraint penalty F are a function of the level set ϕ. We then optimize the structure using a steepest descent method. Using the chain rule, the gradient of the penalty function F is given by

$$\frac{dF}{d\phi} = \frac{dF}{dz}\frac{dz}{d\phi} = \frac{dF}{dz}\frac{d}{d\phi}m(\phi) \tag{S.18}$$

since $z = m(\phi)$. The majority of the computational cost comes from computing the gradient dF/dz.

As described in the main text, we evolve the level set function ϕ by advecting it with a velocity field $v \in \mathbb{R}^{U \times V}$. To implement gradient descent, we set the velocity field to be equal to the gradient of the penalty function:

$$v = \frac{dF}{d\phi}. \tag{S.19}$$

1.6 Computing gradient of penalty function F

We now consider how to efficiently compute the gradient of the penalty function F with respect to the permittivity z, which can be written using (S.15) as

$$\frac{dF}{dz} = \sum_{i=1}^{M} \frac{d}{dz} f_i(x_i). \tag{S.20}$$

Although f_i is not a holomorphic function since $f_i : \mathbb{C}^n \to \mathbb{R}$, we can compute df_i/dz using the expression

$$\frac{d}{dz} f_i(x_i) = 2\,\mathrm{Re}\left(\frac{\partial f_i}{\partial x_i}\frac{dx_i}{dz}\right) \tag{S.21}$$

where we have taken the Wirtinger derivative of f_i [4]. The Wirtinger derivative with respect to some complex variable $w = u + iv$ is defined as

$$\frac{\partial}{\partial w} = \frac{1}{2}\left(\frac{\partial}{\partial u} - i\frac{\partial}{\partial v}\right). \tag{S.22}$$

Using this definition, the Wirtinger derivatives $\partial f_i/\partial x_i$ are given by

$$\frac{\partial f_i}{\partial x_i} = \sum_{j=1}^{N_i} \frac{\partial}{\partial x_i} I_+\left(\left|c_{ij}^\dagger x_i\right| - \alpha_{ij}\right) + \frac{\partial}{\partial x_i} I_+\left(\beta_{ij} - \left|c_{ij}^\dagger x_i\right|\right) \tag{S.23}$$

where

$$\frac{\partial}{\partial x_i} I_+\left(\left|c_{ij}^\dagger x_i\right| - \alpha_{ij}\right) = \frac{1}{2}\frac{\left(c_{ij}^\dagger x_i\right)^*}{\left|c_{ij}^\dagger x_i\right|} c_{ij}^\dagger \cdot \begin{cases} 0, & |c_{ij}^\dagger x_i| - \alpha_{ij} \geq 0 \\ \dfrac{q}{a}\left||c_{ij}^\dagger x_i| - \alpha_{ij}\right|^{q-1}, & \text{otherwise} \end{cases} \tag{S.24}$$

$$\frac{\partial}{\partial x_i} I_+\left(\beta_{ij} - \left|c_{ij}^\dagger x_i\right|\right) = \frac{1}{2}\frac{\left(c_{ij}^\dagger x_i\right)^*}{\left|c_{ij}^\dagger x_i\right|} c_{ij}^\dagger \cdot \begin{cases} 0, & \beta_{ij} - |c_{ij}^\dagger x_i| \geq 0 \\ \dfrac{q}{a}\left|\beta_{ij} - |c_{ij}^\dagger x_i|\right|^{q-1}, & \text{otherwise.} \end{cases} \tag{S.25}$$

Here, we have used the identity

$$\frac{\partial}{\partial u}|u| = \frac{u^*}{2|u|}. \tag{S.26}$$

Next, we consider how to take the derivative of the electric fields x_i with respect to the permittivity z. If we take the derivative of the discretized Maxwell's equations (S.6) with respect to z, we obtain

$$\begin{aligned} D\frac{dx_i}{dz} - \omega_i^2 \operatorname{diag}(x_i) - \omega_i^2 \operatorname{diag}(z)\frac{dx_i}{dz} &= 0 \\ \left(D - \omega_i^2 \operatorname{diag}(z)\right)\frac{dx_i}{dz} &= \omega_i^2 \operatorname{diag}(x_i) \\ A_i(z)\frac{dx_i}{dz} &= -B_i(x_i) \end{aligned} \tag{S.27}$$

where we have used our definitions of A_i and B_i from (S.8). Rearranging, we find the derivative to be

$$\frac{dx_i}{dz} = -A_i^{-1}(z)B_i(x). \tag{S.28}$$

We obtain our final expression for df_i/dz by subsitututing (S.28) into (S.21):

$$\begin{aligned} \frac{d}{dz}f_i(x_i) &= 2\operatorname{Re}\left(\frac{\partial f_i}{\partial x_i}\frac{dx_i}{dz}\right) \\ &= 2\operatorname{Re}\left(-\frac{\partial f_i}{\partial x_i}A_i^{-1}(z)B_i(x_i)\right) \\ &= 2\operatorname{Re}\left(-\left(A_i^{-\dagger}(z)\frac{\partial f_i}{\partial x_i}^\dagger\right)^\dagger B_i(x_i)\right). \end{aligned} \tag{S.29}$$

Since A_i and B_i are large $n \times n$ matrices, we have rearranged the expression in the final step to require only a single matrix solve rather than n solves. This method for reducing the computational cost of computing gradients is known as *adjoint sensitivity analysis*, and is described in detail elsewhere [5].

The cost of computing dF/dz is dominated by the cost of solving Maxwell's equations. For each input mode $i = 1 \ldots M$, we need to solve both the *forward* problem $x_i = A_i^{-1} b$ to find the electric field x_i, and the *adjoint* problem $A_i^{-\dagger}(z) \frac{\partial f_i}{\partial x_i}^\dagger$ from equation (S.29). Both the forward and adjoint problems can be solved by any standard Maxwell's equation solver [6, 7, 8]. We use a graphical processing unit (GPU) accelerated implementation of the finite-difference frequency-domain (FDFD) method [9, 10].

2 Level set implementation

2.1 Curvature limiting

In the main text, we wrote that we implement curvature limiting by evolving the level set function ϕ with

$$\phi_t - b(\kappa)\kappa \, |\nabla \phi| = 0 \tag{S.30}$$

using the weighting function

$$b(\kappa) = \begin{cases} 1 & \text{for } |\kappa| > \kappa_0 \\ 0 & \text{otherwise.} \end{cases} \tag{S.31}$$

In practice, this has terrible convergence since the weighting function falls off infinitely sharply as the local curvature crosses κ_0. To improve the behaviour of our PDE, we actually use a smoothed weighting function

$$b(x, y) = \exp\left(-\kappa_0^2 \, d^2(x, y)\right), \tag{S.32}$$

where $d(x, y)$ is the Euclidean distance to the nearest element in the set Ω,

$$d(x, y) = \inf_{(\hat{x},\hat{y})\in\Omega} \|(x, y) - (\hat{x}, \hat{y})\|. \tag{S.33}$$

We choose $\Omega = \{(x, y)|\kappa(x, y) > \kappa_0\}$ to be the set of points with a local curvature greater than our threshold κ_0. The distance function $d(x, y)$ can be efficiently computed using the Euclidean distance transform commonly included in image processing libraries.

2.2 Numerical implementation

In our design algorithm, we apply gradient descent using the partial differential equation

$$\phi_t + v(x, y) \, |\nabla \phi| = 0 \tag{S.34}$$

where $v(x, y)$ is the local velocity, and apply curvature limiting with equation S.30. We spatially discretize equation S.34 using Godunov's scheme, and equation S.30 using central differencing, as is common practice [11]. We discretize in the time dimension using Euler's method.

To ensure that our level set equations remain well behaved, we regularly reinitialize ϕ to be a signed distance function [11], where $|\nabla \phi| \approx 1$. Most reinitialization schemes, however, result in subtle shifts in the interface locations, which can cause optimization to fail. We use Russo and Smerka's reinitialization scheme to avoid these issues [12].

3 Additional characterization of 1×3 splitter

3.1 Fabrication robustness

To obtain a better understanding of the fabrication robustness of our 1×3 splitter, we simulated the device for a range of over-etching and under-etching errors, which correspond to lateral growth or shrinkage of the design. We have presented the results in figure S1.

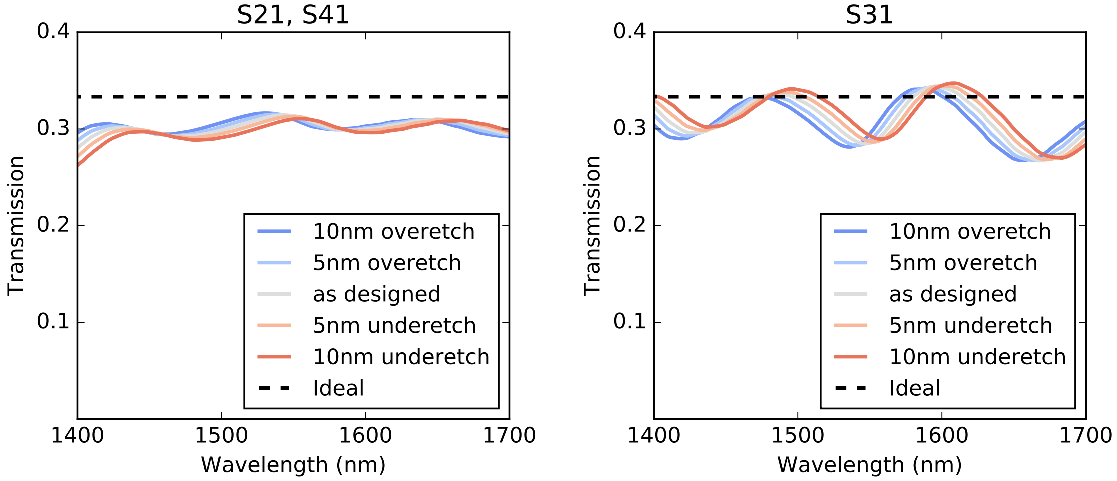

Figure S1: Simulated transmission spectra of the 1×3 splitter for a range of over-etching and under-etching errors, calculated using finite-difference time-domain (FDTD) simulations. For over-etching and under-etching of 10 nm, the device performance is not impacted except for slight spectral shifts.

3.2 Backreflections

We also simulated the return loss for our 1×3 splitter, which we present in figure S2. The backreflections into the fundamental TE_{10} mode of the input waveguide are < 23 dB over the operating bandwidth of our device. Backreflections into the TE_{20} mode are zero due to reflection symmetry in our device in the horizontal direction, and mode conversion to TM modes is impossible due to reflection symmetry of our structure in the vertical direction. Finally, scattering into higher order waveguide modes is negligible since the input and output waveguides are close to single-mode. Thus, backreflections into the input waveguide comprise only a small fraction of the total losses.

References

[1] Miller, D. A. B. All linear optical devices are mode converters. *Opt. Express* **20**, 23985 – 23993 (2012).

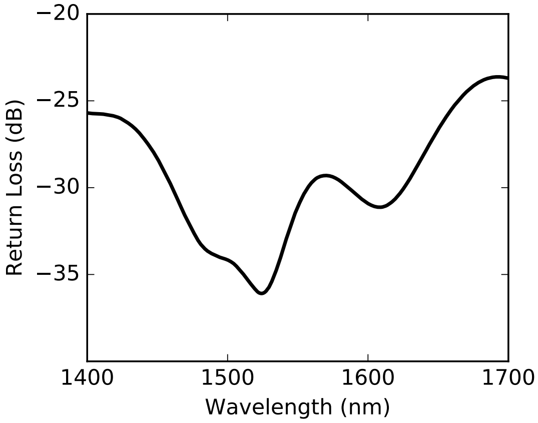

Figure S2: Simulated return loss of the 1×3 splitter back into the fundamental mode of the input waveguide.

[2] McIsaac, P. R. Mode orthogonality in reciprocal and nonreciprocal waveguides. *IEEE Trans. Microw. Theory Techn.* **39**, 1808–1816 (1991).

[3] Boyd, S. & Vandenberghe, L. *Convex Optimization* (Cambridge University Press, Cambridge, U.K., 2004).

[4] Sorber, L., Barel, M. V. & Lathauwer, L. D. Unconstrained optimization of real functions in complex variables. *SIAM Journal on Optimization* **22**, 879–898 (2012).

[5] Giles, M. B. & Pierce, N. A. An introduction to the adjoint approach to design. *Flow, Turbulence and Combustion* **65**, 393–415 (2000).

[6] Nikolova, N. K., Tam, H. W. & Bakr, M. H. Sensitivity analysis with the fdtd method on structured grids. *IEEE Trans. Microw. Theory Tech.* **52**, 1207–1216 (2004).

[7] Lalau-Keraly, C. M., Bhargava, S., Miller, O. D. & Yablonovitch, E. Adjoint shape optimization applied to electromagnetic design. *Opt. Express* **21**, 21693 – 21701 (2013).

[8] Niederberger, A. C. R., Fattal, D. A., Gauger, N. R., Fan, S. & Beausoleil, R. G. Sensitivity analysis and optimization of sub-wavelength optical gratings using adjoints. *Opt. Express* **22**, 12971 – 12981 (2014).

[9] Shin, W. & Fan, S. Choice of the perfectly matched layer boundary condition for frequency-domain Maxwell's equations solvers. *J. Comput. Phys.* **231**, 3406 – 3431 (2012).

[10] Shin, W. & Fan, S. Accelerated solution of the frequency-domain Maxwell's equations by engineering the eigenvalue distribution. *Opt. Express* **21**, 22578 – 22595 (2013).

[11] Osher, S. & Fedkiw, R. *Level Set Methods and Dynamic Implicit Surfaces* (Springer, New York, U.S.A., 2003).

[12] Russo, G. & Smereka, P. A remark on computing distance functions. *J. Comput. Phys.* **163**, 51 – 67 (2000).
